# Impact of mutations in *PRLR* for hair weight, circulating prolactin concentrations, feed intake, and growth of beef cattle in a hot and humid climate

**DOI:** 10.3389/fendo.2026.1907038

**Published:** 2026-07-24

**Authors:** Camila J. Cuellar, Daniella Heredia, Jose L. Infante Cangrejo, Quinn A. Hoorn, Abigail J. Sproull, Mauro Venturini, Federico Tarnonsky, Samuel Hincapié, Nicolas DiLorenzo, Angela M. Gonella-Diaza, Erin L. Oberhaus, Peter J. Hansen

**Affiliations:** 1 Department of Animal Sciences, University of Florida, Gainesville, FL, United States; 2 North Florida Research and Education Center, University of Florida, Marianna, FL, United States; 3 School of Animal Sciences, Louisiana Agricultural Experiment Station, LSU Ag Center, Baton Rouge, LA, United States

**Keywords:** beef cattle, feed efficiency, growth, heat stress, prolactin receptor, slick mutation

## Abstract

**Background:**

In cattle, mutations in the prolactin receptor gene (*PRLR*) causing loss of tyrosine residues important for JAK-STAT signaling result in a short hair coat and increased ability to regulate body temperature during heat stress. Such mutations, referred to as slick mutations, occur naturally and have been introduced into cattle by gene editing.

**Aim:**

Consequences of inheritance of various mutations in *PRLR* for growth in beef cattle raised in a hot, humid climate (Florida) were evaluated.

**Methods:**

Beef cows (predominantly Angus) were inseminated with semen from a single Angus sire mosaic for slick mutations in *PRLR*. Calves were born from November to January, raised with their dams until weaning on July 14 at 169 to 245 days of age, and then placed in a 56-day feed efficiency trial (August 25–October 21) in which individual feed intakes and body weights were recorded.

**Results:**

There were a variety of genotypes for *PRLR.* The number of calves at weaning included 64 that were heterozygous for one of five mutations causing a slick phenotype (SLICK), 6 wild-type calves without a mutation affecting the prolactin receptor (WT), and 7 calves heterozygous for a large deletion in the *PRLR* gene resulting in a functional knockout (NULL). Hair weights were greatest for NULL and, after 3 months of age, least for slick. While body weight was not affected by *PRLR* genotype through weaning, average daily gain, dry matter intake, and body weight at the end of the trial adjusted for starting weight were significantly greatest for SLICK, intermediate for WT, and least for NULL. Residual feed intake was significantly greater (i.e., lower efficiency) for SLICK than WT or NULL. Vaginal temperatures during the feed efficiency trial were higher for NULL than SLICK heifers.

**Conclusions:**

Genotype for *PRLR* has important effects on ability for post-weaning growth. These effects are most likely related to differences in hair growth and ability to regulate body temperature during hot weather.

## 1 Introduction

Cattle are homeotherms, and environmental conditions such as high air temperatures, intense solar radiation, and high humidity cause heat stress that exacerbates body temperature regulation and can lead to depressed milk yield and growth, reduced sexual behavior and fertility, compromised fetal development, and death (1). The impact of heat stress on cattle production, as well as on production of other food animals, is expected to grow because of projected increases in Earth’s surface temperature (2). Among the useful strategies for reducing effects of heat stress on cattle is to improve genetic resistance by identification of alleles conferring thermotolerance in populations of cattle adapted to hot climates followed by use of conventional or gene-editing techniques to introgress those alleles into populations of cattle that are not thermotolerant (1, 3).

To date, the prolactin receptor (*PRLR*) is one of the few genes in cattle with identified mutations that confer thermotolerance. Six separate single-nucleotide polymorphisms in the gene have been identified that result in a truncation of the encoded receptor protein at the C-terminal end leading to the loss of the JAK/STAT binding site (4–6). These mutations are dominant and are called slick mutations because cattle inheriting either one or two copies of the mutation exhibit a sleek, short hair coat (7). Cattle with even one slick allele exhibit superior regulation of body temperature during heat stress. This is the case for both dairy cattle (7–12) and beef cattle (7, 13). Lactating cows inheriting slick alleles from Senepol or Carora cattle had increased milk yield in hot climates (7, 9, 14). Inheritance of a slick allele was associated with increased yearling weight of Senepol beef cattle in Brazil (15).

Recently, gene editing was used to introduce a slick mutation to Jersey and Angus cattle (16). Gene-edited animals with two copies of a slick allele of *PRLR* exhibited superior ability to regulate body temperature, heavier body weights, and increased scrotal circumference compared with non-slick cattle. There was no evidence of negative effects of the mutation on carcass characteristics or male reproductive function.

The importance of the prolactin signaling system for regulation of hair growth and thermoregulation was further demonstrated by a study evaluating characteristics of predominantly Holstein–Friesian females sired by a bull with a loss-of-function mutation in the prolactin gene (*PRL*) (4). These animals produced excessive amounts of hair and were less able to regulate body temperature during heat stress than control females. This result also leads to the conclusion that slick mutations in *PRLR* represent gain-of-function mutations, at least with certain characteristics, because slick mutations cause phenotypes opposite to those of loss-of-function mutations for *PRL* including reduced hair growth and enhanced ability to regulate body temperature during heat stress.

In the current experiment, an Angus bull produced by gene editing that was chimeric at the *PRLR* locus was used to generate offspring that inherited one of several alleles of the receptor gene including the wild-type allele associated with normal hair growth, several alleles with a slick phenotype, and alleles causing a functional knockout of the receptor. Generation of these cattle allowed examination in half-siblings of the consequences of mutations in *PRLR* for hair growth, regulation of prolactin secretion, and body weight gain for cattle raised in a hot climate. Results confirm the importance of the prolactin receptor signaling pathway for regulation of hair growth, body weight, and average daily gain and demonstrate that knockout of a single copy of *PRLR*, like for loss-of-function mutations in *PRL*, leads to excessive hair growth and reduced body weight gain.

## 2 Materials and methods

### 2.1 Animal care

Cows and their calves were housed at the University of Florida North Florida Research and Extension Center in Marianna, Florida (30°51′03″ N, 85°09′54″ W), throughout the research trial.

### 2.2 Generation of calves from a bull chimeric for the prolactin receptor gene

The sire used to produce calves with modifications in *PRLR* was a red-carrier Angus sire (Stay Cool, Red Angus Association of America registration number 4410061)] who had a mosaic set of genotypes for *PRLR*. The bull was bred by Acceligen (Eagen, MN, USA) from an embryo treated with CRISPR/Cas ribonucleoprotein complexes to produce slick allelic variants of the *PRLR* gene. The resultant mosaic genotypes included 1) a non-edited allele, 2) an allele with an-in frame 3-bp deletion predicted to function as a non-slick phenotype, 3) five different alleles with indel mutations that result in truncation of *PRLR* that would produce the slick phenotype, and 4) a large deletion that would result in a non-functional *PRLR* allele (null allele).

A total of 173 cows [Angus crossbreds (n=97), Brangus (n=43), Simmental–Angus crossbreds (n=15), and Braford (n=18) were artificially inseminated using frozen-thawed conventional semen from the chimeric bull during the spring breeding season (February 15 to April 19, 2024). Cows were synchronized using a timed artificial insemination protocol based on the 7-day CO-Synch + CIDR estrous synchronization protocol. On day −9, cows received 100 μg gonadotropin-releasing hormone (GnRH; Cystorelin, Boehringer Ingelheim, Duluth, GA, USA) administered, i.m., along with insertion of a controlled internal drug release device containing 1.38 g progesterone (CIDR; Zoetis, Parsippany-Troy Hills, NJ, USA). Each CIDR device was removed on day −2, and each cow received 25 mg prostaglandin F_2_α (Lutalyse; Zoetis), i.m. Another i.m. injection of 100 μg GnRH was given with insemination 56 ± 2 h later (day 0). Pregnancy diagnosis was performed at 30 days of gestation via transrectal ultrasonography using a MyLabDelta ultrasound (Esaote, Genoa, Italy).

Cows were managed in three breeding groups. Cows diagnosed as non-pregnant on day 30 after the first insemination were resynchronized and subjected to a second timed artificial insemination using the same protocol as listed above. Pregnant cows were subsequently maintained together under uniform management conditions in Bahia grass (*Paspalum notatum*) pastures. Calving occurred between November 2024 and January 2025. A total of 83 calves were produced by artificial insemination. Of these, three were stillborn and one calf died postnatally due to injury during sample collection. Ear punches were obtained from the remaining calves and used for genotyping.

Genotyping of the calves was performed using genotyping services provided by Acceligen. All calves inherited one wild-type allele of *PRLR* from their dam. A total of 66 calves were heterozygous for one of five mutations causing a slick phenotype (SLICK), 7 calves were heterozygous for the non-functional allele (NULL), and 6 calves did not have a mutation affecting the prolactin receptor (wild type; WT). Distribution of calves by sex is shown in **Table 1**. Note that one SLICK heifer calf was excluded from the experiment because it was very small in size (weaning weight = 95 kg). In addition, one SLICK bull calf died postnatally during sample collection at 5 months of age so the number of SLICK animals in the experiment was 65 until 5 months of age and 64 after 5 months of age.

**Table 1:** Number of calves of each genotype class^a^.

Genotype^a^	Hair coat	Total no.	No. of bulls	No. of heifers
SLICK	slick	66^b^	37	29
WT	normal	6	4	2
NULL	hairy	7	1	6

a SLICK and NULL animals were heterozygous with the other allele being wild type.

b One heifer calf was excluded from the experiment because it was very small in size (weaning weight = 95 kg). In addition, one bull calf died postnatally during sample collection at 5 months of age so the number of slick animals in the experiment was 65 until 5 months of age and 64 after 5 months of age.

### 2.3 Management of cows and calves from birth through weaning

Calves remained with their dams until weaning on July 15, 2025, at approximately 7 months of age (mean = 209 days; range of 169–244 days). Calves and dams were maintained on Bahia grass pasture *ad libitum* throughout the pre-weaning period. Body weight at birth was recorded using an electronic scale. Body weight, hip height, hair weight, and blood collection via jugular venipuncture for measurements of plasma prolactin concentrations were collected at approximately 1 (average age = 36 days, range 15–53 days), 3 (average age = 81 days, range 65–100 days), and 5 months of age (average age = 147 days, range 126–160 days), and at weaning (average age = 209, range 169–244 days). Male calves were castrated at ~3 months of age (average age = 81 days, range 65–100 days) and testes collected and weighed.

Body weight and hip height measurements were taken while the animals were in a holding chute (Silencer Squeeze Chute; Moly Mfg. Inc., Lorraine, KS, USA). Body weight was measured using an electric scale (Tru-Test Livestock Inc., Mineral Wells, TX, USA), and hip height was measured using an aluminum measuring stick. Adjusted 205-day weaning weight was calculated using the formula [(weaning weight−birth weight)/days of age at weaning] × 205 + birth weight.

Hair growth was assessed by shaving a 7.5 cm × 5 cm area located between the hook and pin bones on the right side of the pelvis. A new area that had not been shaved before was selected for every time point. The removed hair was collected and placed into a pre-weighed plastic bag and weighed to determine hair weight.

Blood samples were collected via jugular venipuncture into tubes containing ethylenediaminetetraacetic acid (BD Biosciences, East Rutherford, NJ, USA). Samples were kept on ice until centrifugation at 2,000 × g for 30 min at room temperature. Plasma was then separated and stored at −80°C until further analysis. Concentrations of prolactin were measured by radioimmunoassay using an anti-sheep prolactin antibody (17). Parallelism was established between bovine plasma and the ovine prolactin standard curve. Recovery of prolactin after spiking a pool of bovine plasma with 20, 50, and 100 ng prolactin ranged from 79% to 89%. Intra- and interassay CVs and the limit of detection were 3%, 10%, and 0.3 ng/mL, respectively.

### 2.4 Feed efficiency trial

Offspring were enrolled in a 56-day feed efficiency trial when animals were at an average age of 400 days. After an adaptation period of 2 weeks, the feed efficiency trial was performed from August 25 to October 21, 2025. Steers and heifers were randomly balanced by sex and genotype to four pens of 19–20 cattle each for 56 days for feed efficiency assessment. All cattle were fed the same diet during this period (**Supplementary Table 1**). Steers and heifers were weighed on two consecutive days at the beginning (day −1 and day 0) and at the end (day 55 and day 56) of the feed efficiency trial to calculate the average initial and final body weight, respectively. Another body weight was measured on day 28 of the trial. Individual feed intake was collected with the SmartFeed intake monitoring system (C-Lock Inc., Rapid City, SD, USA). Each pen had two bunks that received feed to measure individual intake based on radio-frequency identification technology. Feed was delivered every morning at 0800 H using a horizontal mixer wagon (Kuhn Knight 1142 Helix; Kuhn North America, Inc., Brodhead, WI, USA). Feed samples were collected once weekly for dry matter and chemical analyses. Feed samples were dried in a forced-air oven at 55 °C for 72 h. At the end of the experiment, feed samples per week were ground in a Willey mill (Arthur H. Thomas Co., Philadelphia, PA, USA) to pass a 2-mm screen and composite. This composite sample was analyzed for nutritional composition by a commercial laboratory (Dairy One Forage Laboratory, Ithaca, NY) through wet chemistry procedures for concentrations of crude protein, neutral detergent fiber, ether extract, lignin, and starch. The ingredient composition and nutrient profile of the diet are presented in **Supplementary Table 1**.

Dry matter intake and average daily gain were assessed. Initial body weight was calculated as the average of the unshrunk body weight collected on day −1 and day 0. Final body weight was calculated as the average of the unshrunk body weight on day 55 and day 56. Body weight change was computed as final minus initial body weight. Daily gain was computed as body weight change divided by days on feed. Residual feed intake was calculated as the difference between actual (measured) intake and expected (predicted) intake of each individual (18). Predicted intake was calculated through the regression of actual (measured) dry matter intake on average daily gain and mid-metabolic body weight (MBW; mid-test body weight^0.75^) using PROC REG (SAS 9.4 Inst. Inc., Cary, NC), according to the following model: Y = β0 + β1 X1 + β2 X2 + ϵ, where Y is expected dry matter intake, β0 is the equation intercept, β1 and β2 are the coefficients of the regressors, X1 is MBW, X2 is average daily gain, and ϵ is the residual. The adjusted R^2^ for this model was 0.4861, and the mean square error was 1.673.

### 2.5 Vaginal temperatures

During the 2-week adaptation period of the feed efficiency trial, vaginal temperature was measured at 10-min intervals for 5 days using an iButton 1922L (Maxim Integrated, San Jose, CA, USA) placed inside a blank (i.e., no progesterone) controlled internal drug-releasing (CIDR) device (Zoetis, Kalamazoo, MI, USA) that was inserted into the vagina. The procedure was as described elsewhere (19). Each iButton was set to 0.0625 °C accuracy, inserted intravaginally of the morning of day 1, and removed on the morning of day 5. The process was done during two replicates of 5 days each (August 4 to 8 and August 18 to 22, 2025) with separate heifers in each replicate. The mean and standard deviation for average temperature-humidity index (THI) was 77.3 and 0.75 for replicate 1 and 78.6 and 1.5 for replicate 2. Note that there were only two WT heifers and the iButton failed for one WT heifer. Data were available for 20 SLICK, 1 WT, and 5 NULL heifers. Accordingly, statistical analysis was performed for SLICK and NULL only.

### 2.6 Measurement of environmental variables

Weather data during the trial for Marianna, Florida, from November 2024 to November 2025 were obtained from https://fawn.ifas.ufl.edu/. Average daily dry bulb temperature was obtained from the dry bulb temperature recorded 2 m above the ground. The temperature-humidity index was calculated using the following equation (20):


THI=(1.8 × Tdb+32)−[(0.55 − 0.0055 × RH) × (1.8 × Tdb−26.8)]

where Tdb=dry bulb temperature (°C) and RH=relative humidity.

### 2.7 Statistical analysis

Data were analyzed using the Statistical Analysis System version 9.4 (SAS Institute, Cary, NC, USA). Continuous data at each collection date were analyzed by analysis of variance using the GLM procedure of SAS. Data on prolactin concentrations were log-transformed before analysis. The statistical models included the genotype (SLICK, WT, and NULL), sex (male and female), and breeding group (1, 2, and 3), and interactions as class effects and with age as a covariate. Effects of genotype were partitioned into the following two orthogonal contrasts: SLICK vs. WT and NULL vs. SLICK + WT. Non-significant effects in which the F was < 2.0 were removed from the model to improve parsimony. Data on hair weight and prolactin concentrations were also analyzed by PROC GLIMMIX to determine the effect of age. The statistical model included effects of genotype, group, sex, age, and genotype × age and with animal nested within group–sex–genotype as a random effect. Data on vaginal temperature were analyzed by PROC GLIMMIX with main effects of genotype (SLICK vs. NULL), time nested within day, replicate, and all interactions as fixed effects and with heifer nested within genotype and replicate as a random effect.

## 3 Results

### 3.1 Environmental conditions during the experiment

**Figure 1:**
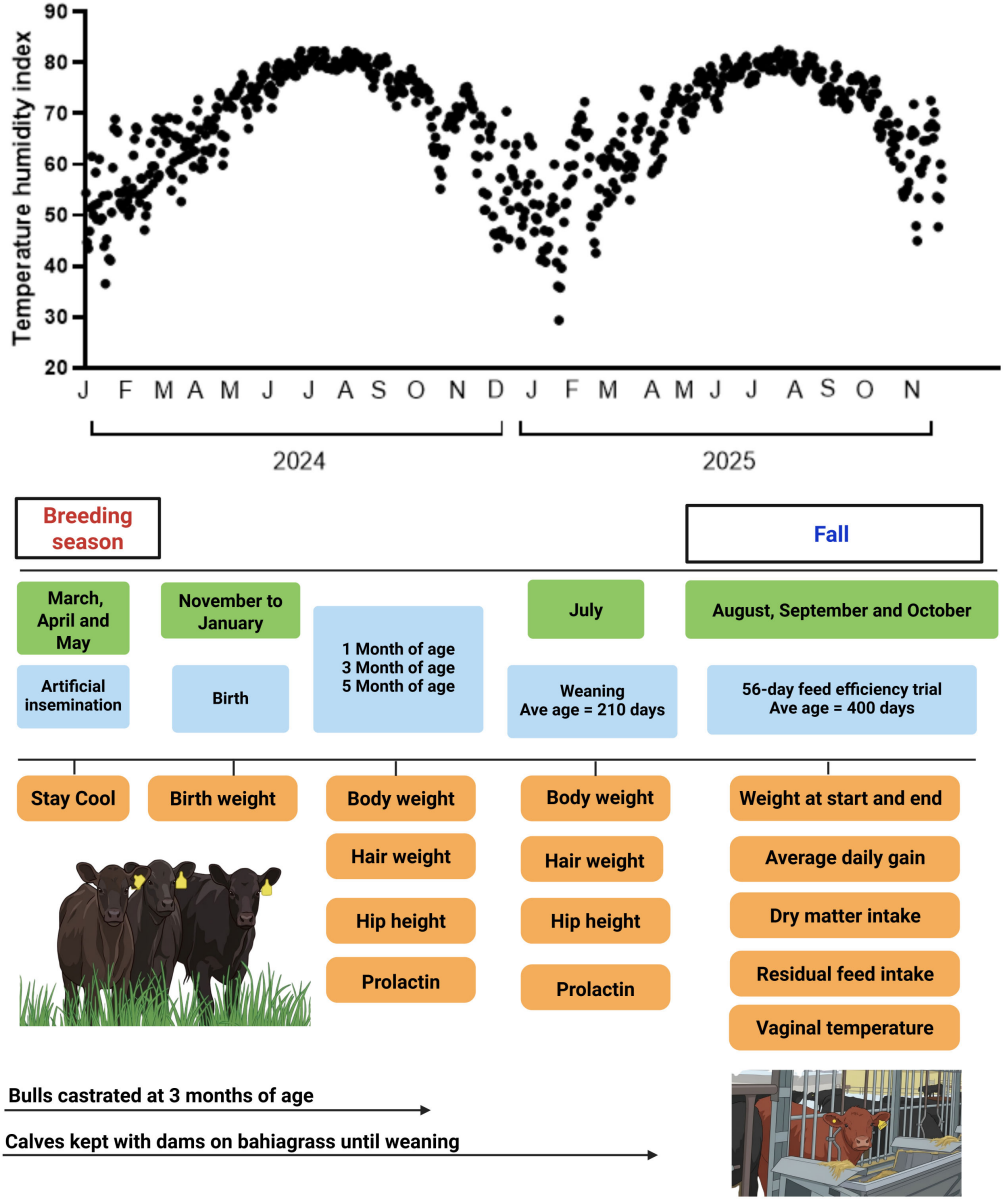
Environmental conditions during the experiment. The top panel shows daily average temperature-humidity index, and the bottom panel presents an overview of the timing of the experimental procedures.

Data on average daily THI during the experiment are shown on **Figure 1**. Calves born from the chimeric bull were born November to January 2024 at a time when THIs were low. As calves advanced in age, environmental temperatures increased. Average daily THI above 70, a value associated with negative effects of heat stress (21), were predominant during the months of March through September. Thus, some of the experiment encompassing measurements of preweaning growth and most of the period encompassing postweaning growth was carried out during extended periods of heat stress.

### 3.2 Hair weights and prolactin concentrations

**Figure 2:**
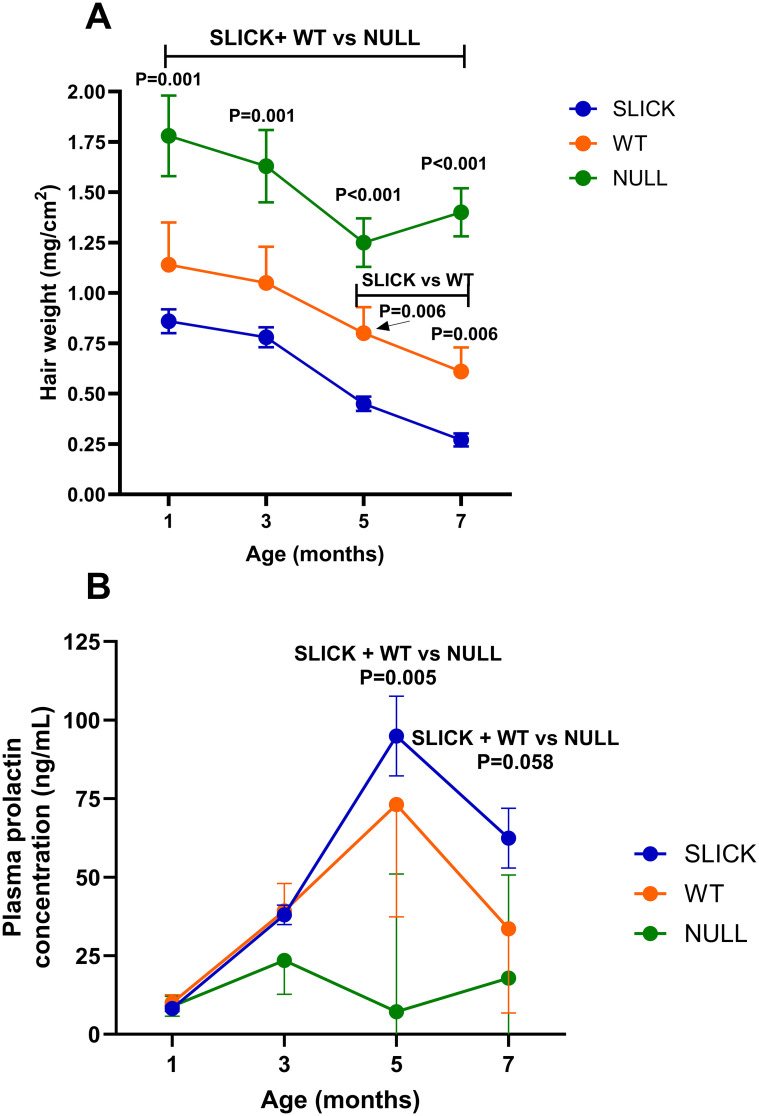
Effects of genotype and age on hair weight **(A)** and plasma concentrations of prolactin **(B)**. Overall, hair weight was affected by genotype (P<0.0001), age (P<0.0001), and the interaction between genotype and age (P = 0.011). Prolactin concentrations were affected by genotype (P = 0.058), age (P<0.0001), and the interaction between genotype and age (P = 0.0007). Effects of genotype at specific months are detailed in the body of each graph. Data are least-squares means ± SEM.

Hair weights were affected by genotype (P<0.0001), age (P<0.0001), and genotype × age (P = 0.011), with hair declining with age in all groups (**Figure 2A**). Subsequently, effects of genotype were analyzed separately at 1, 3, 5, and 7 months of age (i.e., on the date of weaning) using orthogonal contrasts to compare SLICK vs. WT and SLICK + WT vs. NULL. Hair weight in SLICK and WT was significantly less than NULL at all times measured. There were no differences in hair weight between SLICK and WT at 1 and 3 months of age, but hair weight at 5 and 7 months of age was less (P = 0.006) for SLICK than WT at 5 and 7 months of age.

Plasma concentrations of prolactin are shown in **Figure 2B**. Concentrations were affected by genotype (P = 0.058), age (P<0.0001), and the interaction between genotype and age (P = 0.0007). In SLICK and WT calves, concentrations of prolactin increased from 1 to 5 months of age and then declined at 7 months of age. In contrast, concentrations of prolactin remained low throughout the first 7 months of age for NULL calves. Effects of genotype were also analyzed separately at 1, 3, 5, and 7 months of age. There were differences for SLICK + WT vs. NULL at 5 (P = 0.005) and 7 months of age (P = 0.058).

### 3.3 Body weight and hip height at 1, 3, and 5 months of age

**Figure 3:**
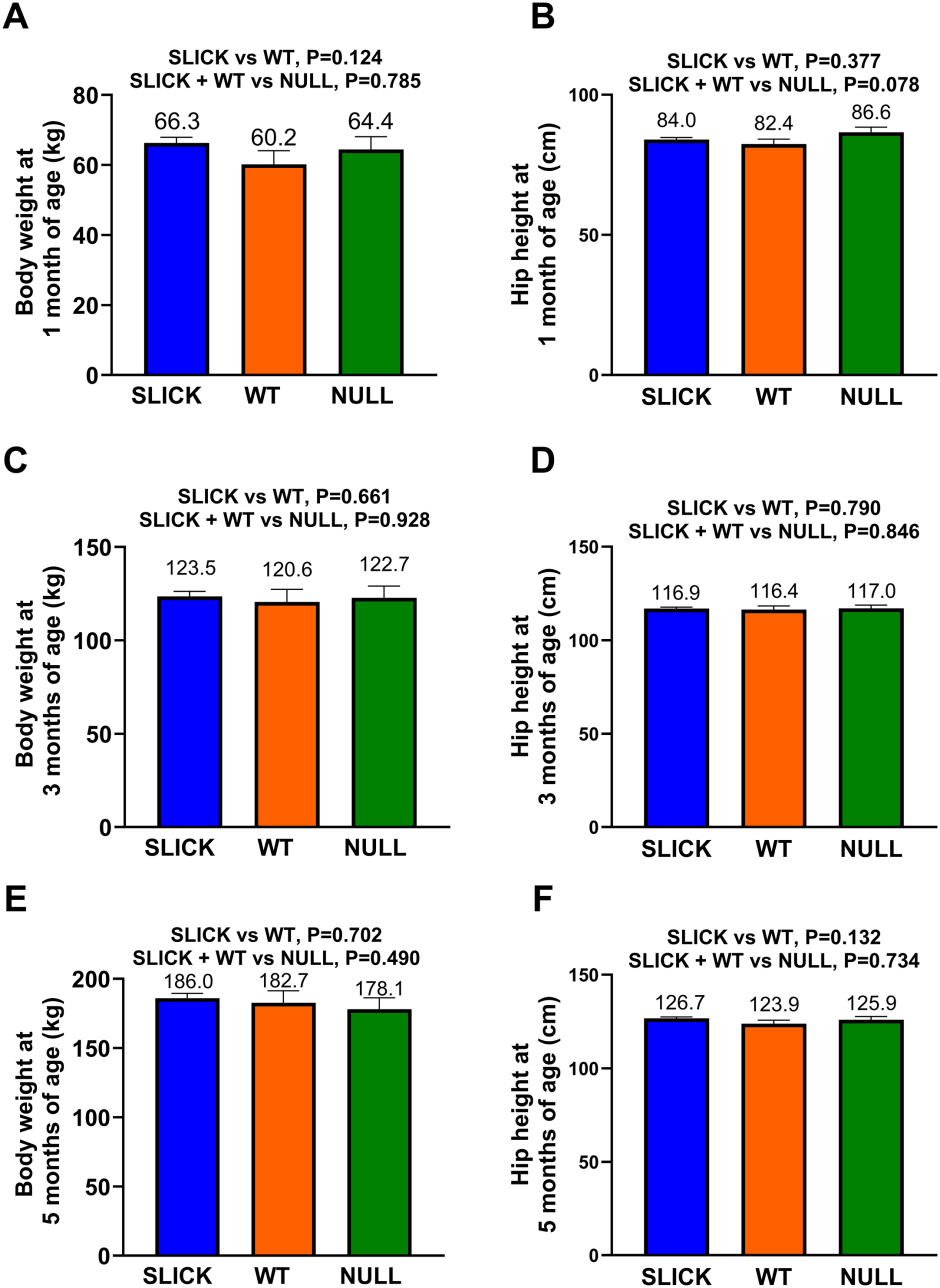
Effects of genotype on body weight and hip height at 1 **(A, B)**, 3 **(C, D)**, and 5 months of age **(E, F)**. Effects of genotype at specific months are detailed in the body of each graph. Data are least-squares means ± SEM.

In general, there were no effects of genotype on body weight or hip height at 1, 3, or 5 months of age (**Figure 3**). The one exception was a tendency for hip height at 1 month of age to be greater in NULL than SLICK + WT (P = 0.078).

### 3.4 Testis weight

Testis weight at castration (average age 81 days) tended to be greater for SLICK than WT (P = 0.074), whereas there was no difference for NULL vs. SLICK + WT (P = 0.362). The least-squares means ± SEM for paired testis weight was 36.7 ± 2.4 g for SLICK, 29.4 ± 4.0 g for WT, and 26.2 ± 7.5 g for NULL. In contrast, the gonadosomatic index (i.e., testis weight per unit body weight) was not affected by the comparison of SLICK vs. WT (P = 0.197) or NULL vs. SLICK + WT (P = 0.402). The least-squares means ± SEM for gonadosomatic index were 0.27 ± 0.02 g/kg for SLICK, 0.23 ± 0.03 g for WT, and 0.21 ± 0.05 g for NULL.

### 3.5 Body weight and hip height at weaning

**Figure 4:**
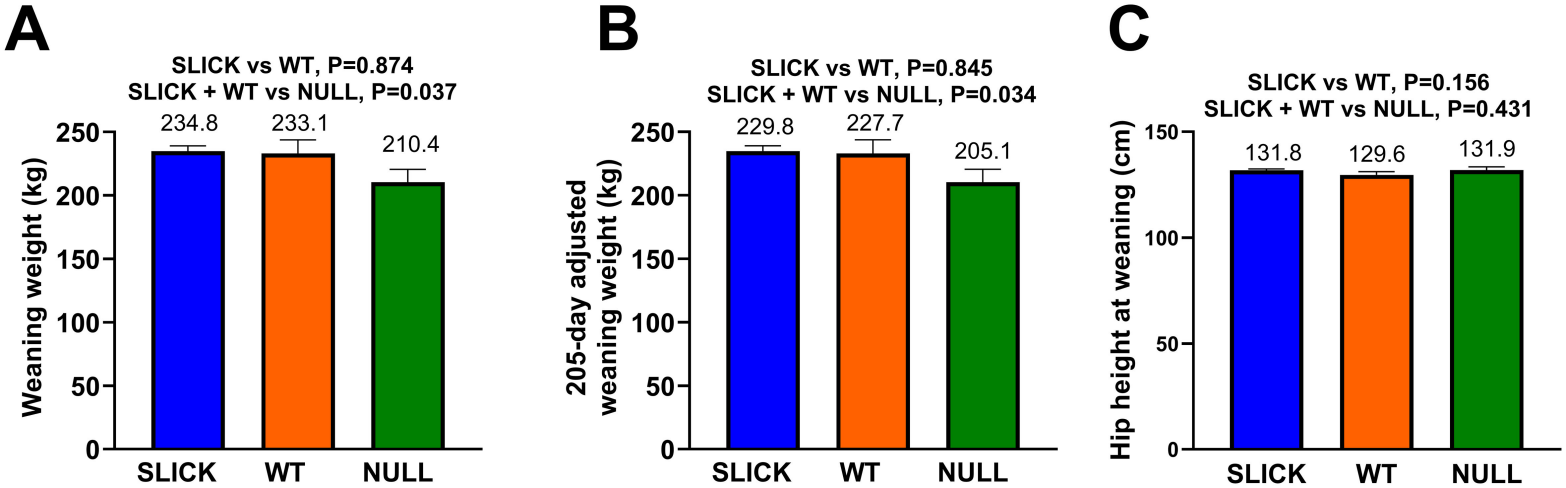
Effects of genotype on body weight at weaning **(A)**, 205-day adjusted body weight **(B)**, and hip height at weaning **(C)**. Effects of genotype at specific months are detailed in the body of each graph. Data are least-squares means ± SEM.

Weaning weight (**Figure 4A**) and 205-day adjusted weaning weight (**Figure 4B**) were not affected by the comparison of SLICK vs. WT, but SLICK + WT had heavier weaning weights (P = 0.037) and 205-day adjusted weaning weights (P = 0.034) than NULL calves. Hip height (**Figure 4C**) was not affected by genotype.

### 3.6 Feed efficiency

**Figure 5:**
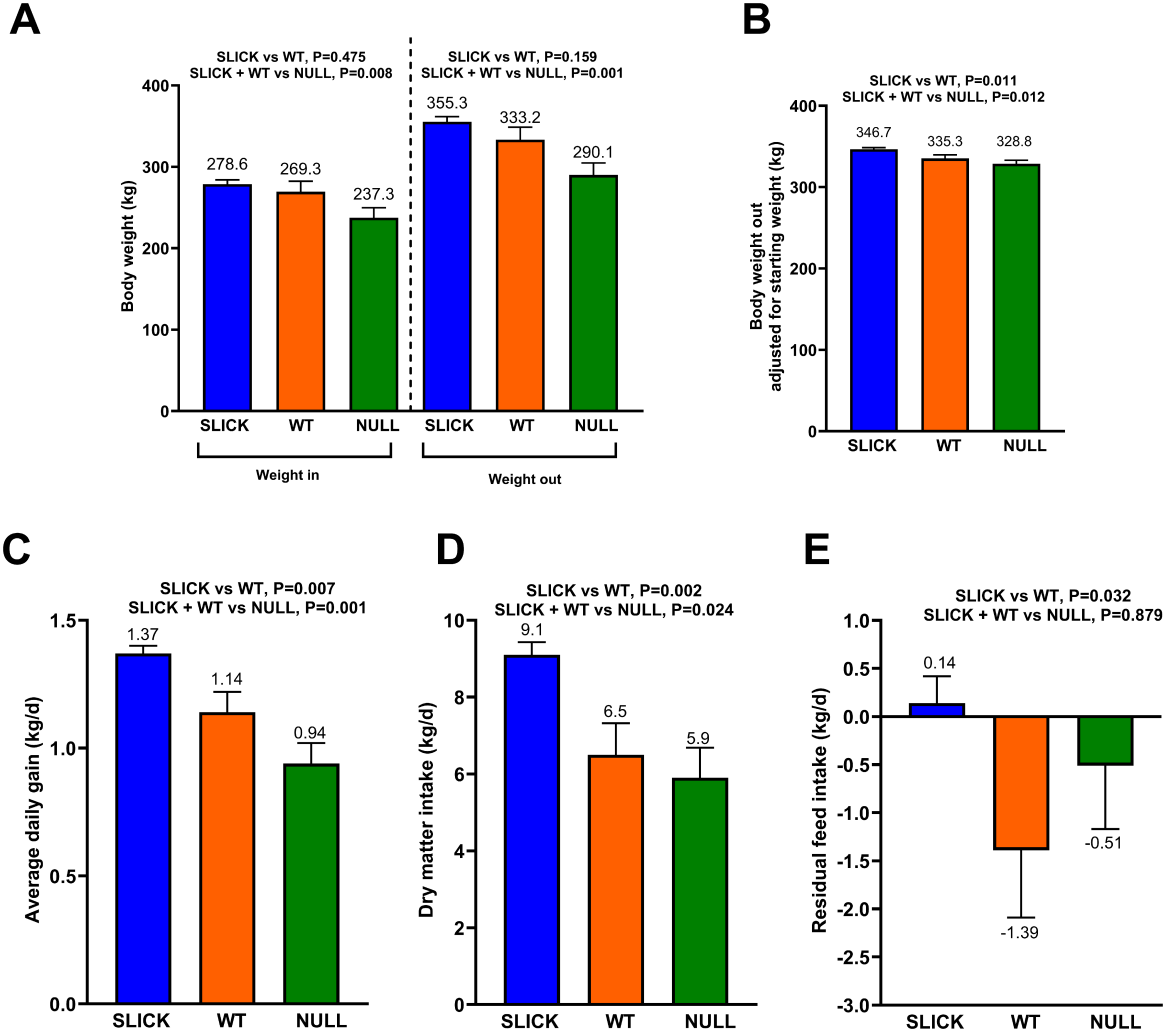
Effects of genotype on body weight at beginning and end of the trial: **(A)**, body weight at the end of the trial adjusted for starting weight **(B)**, average daily gain **(C)**, and dry matter intake **(D)** and residual feed intake **(E)** during the feed efficiency trial at ~400 days of age. Effects of genotype are detailed in the body of each graph. Data are least-squares means ± SEM.

There was no difference in body weight between SLICK and WT at the beginning of the feed efficiency trial (P = 0.475), but NULL calves were lighter than SLICK + WT (P = 0.008; **Figure 5A**). However, after adjusting for starting weight, body weight at the end of the trial was greater for SLICK vs WT (P=0.011) and less for NULL than SLICK + WT (P=0.012; **Figure 5B**). Average daily gain (**Figure 5C**) was greater for SLICK than WT (P = 0.007) and lower for NULL than SLICK + WT (P = 0.001). Similarly, dry matter intake (**Figure 5D**) was greater for SLICK vs. WT (P = 0.002) and lower for NULL vs. SLICK + WT (P = 0.024). Residual feed intake (**Figure 5E**) was greater for SLICK vs. WT (P = 0.032), i.e., SLICK were less feed efficient, but there was no difference between SLICK + WT vs. NULL (P = 0.879).

**Figure 6:**
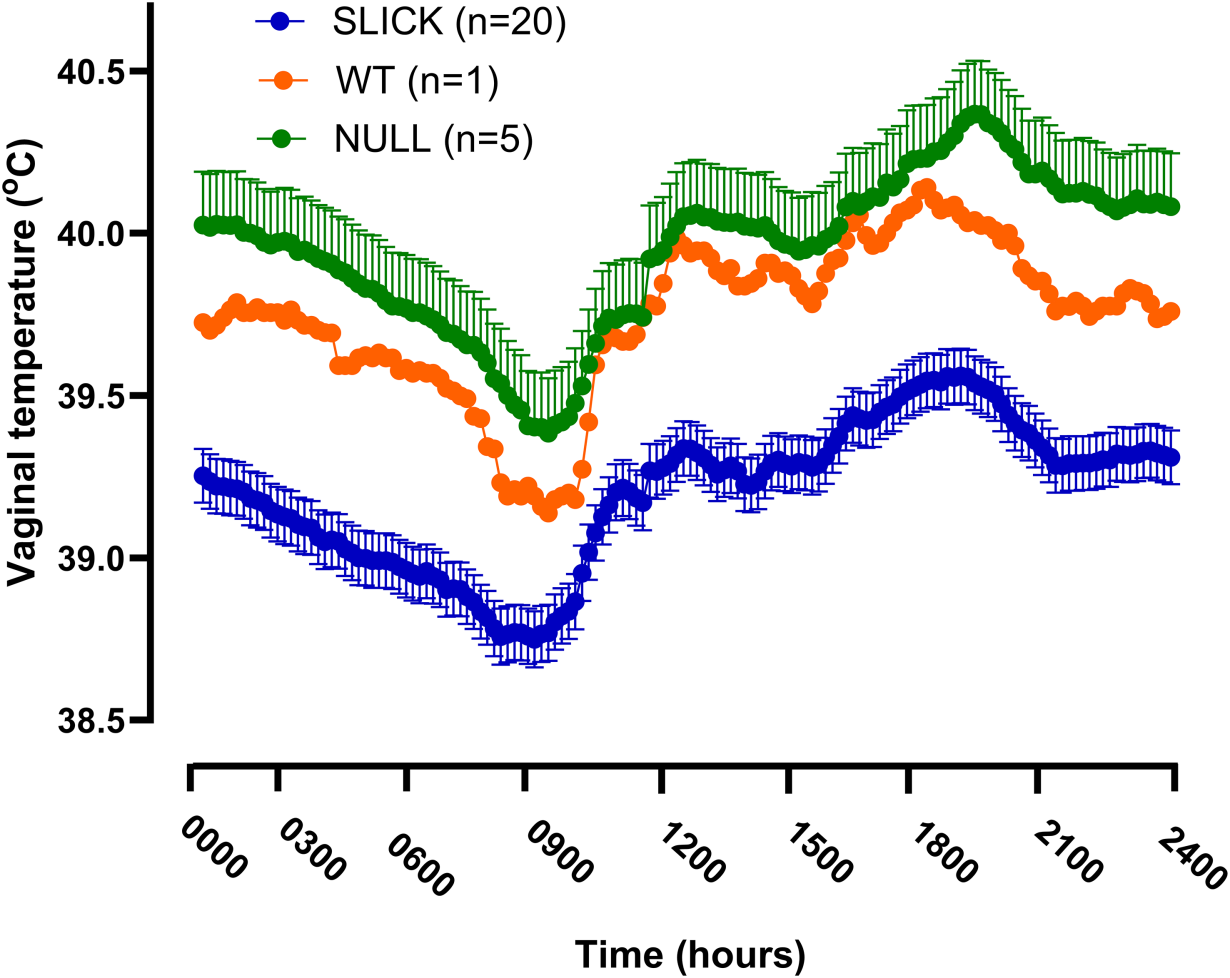
Effects of genotype and time of day on vaginal temperature of heifers measured during the feed efficiency trial. The number of heifers is indicated in the legend. There was only one WT heifer (note the absence of error bars), and this group was excluded from statistical analysis. Vaginal temperature was affected by genotype (SLICK vs NULL) (P = 0.0002), time (P<0.001), date nested within replicate (P<0.0001), time by genotype interaction (P = 0.034), replicate (P<0.001), time by replicate interaction (P<0.001), and genotype by replicate interaction (P<0.0001). Data are least squares means ± SEM.

Vaginal temperature was measured during August for heifers. Results are shown in **Figure 6**. Only one heifer in the WT group was measured because of a technical failure with the iButton in the other WT heifer. Thus, statistical analysis was performed comparing SLICK vs. NULL. Vaginal temperatures were lower for SLICK than NULL (P=0.0002). This was true throughout the day. A genotype × time interaction (P = 0.034) reflected the fact that the magnitude of the difference in vaginal temperature between genotypes was greatest before 0900 and after ~2000 H. There was also a genotype × replicate interaction (P<0.0001) because the difference between NULL and SLICK was greater for replicate 2 (40.42 ± 0.15 for NULL vs. 39.16 ± 0.08 °C for SLICK) than for replicate 1 (39.48 ± 0.15 for NULL vs. 39.24 ± 0.08 °C for SLICK). Note that the average THI was slightly higher for replicate 2 (78.6) than for replicate 1 (77.3).

## 4 Discussion

Mutations in *PRLR* that cause truncation of the C-terminus of the encoded protein give rise to the slick hair phenotype, characterized by a shorter hair coat that enhances heat dissipation and improves the animal’s ability to cope with thermal stress. Notably, inheritance of a single copy of this mutation is sufficient to produce the slick phenotype, which has been linked to improved thermoregulation, milk production, and growth under heat stress conditions. Here, we confirm that the inheritance of a single SLICK allele can improve post-weaning growth in beef cattle raised in hot climates and demonstrate that mutations that render *PRLR* non-functional compromise growth. Both mutations are associated with changes in hair weight consistent with differences in the ability to regulate body temperature. The null mutation in *PRLR* also caused a large decline in circulating prolactin concentrations.

One of the observations of the present experiment was that differences in body weight due to *PRLR* genotype only became apparent as calves became older and entered warmer periods of the year. There were no significant differences between SLICK and WT genotypes until the feed efficiency trial that occurred beginning at ~400 days of age from August to October. During the trial, average daily gain and dry matter intake were higher for SLICK than WT. Body weight at the end of the trial was greater for SLICK than WT when adjusting for starting weight. NULL calves had lower weights than SLICK + WT at weaning and all times thereafter.

The most likely explanation for the delay in effect of genotype on body weight and growth is a combination of increasing incidence of heat stress as animals grew older as well as changes in hair coat. Calves were born in the winter and were not exposed to extensive heat stress until March when they were 3–4 months old. Examination of changes in hair weight with age indicated that all groups had more extensive hair coat at 1 month of age during the winter than at later times and that calves were shedding hair between 1 and 7 months of age. This is particularly true for SLICK and WT calves. As shedding occurred, the absolute amount of hair was reduced in SLICK calves as compared with WT at 5 and 7 months of age. A reduction in hair length is the defining characteristic of slick mutations in *PRLR*. In contrast to SLICK and WT, NULL calves continued to have heavy hair coats through July. Increased hair growth in NULL calves was expected because a loss-of-function mutation in *PRL* also causes excessive hair growth (4). Results here indicate that even loss of a single copy of *PRLR* is sufficient to cause large alterations in pelage phenotype. These differences in hair coat between genotypes are the most likely reason for differences in growth because clipping hair reduced hyperthermia in heat-stressed calves (22, 23).

Prolactin has been implicated in seasonal changes in pelage (24). In cattle, both long day photoperiods (25–27) and heat stress (28) can increase circulating concentrations of prolactin whereas exposure to cold temperatures decreases concentrations (29). Plasma concentrations of prolactin in the present experiment increased over time, coincident with increasing photoperiod and temperature, before declining from 5 to 7 months of age. Concentrations of prolactin were similar between SLICK and WT calves. In an earlier experiment, slick calves had similar concentrations of prolactin as wild-type calves at 221 days of age but greater concentrations at 535 days of age (16). Inheritance of a NULL mutation in *PRLR* blunted temporal changes in prolactin concentrations so that concentrations remained low through weaning.

The finding that prolactin concentrations were lower in NULL calves is counter-intuitive since one would expect that a reduction in functional prolactin receptors would remove positive feedback actions of prolactin on the prolactin inhibitory transmitter dopamine (30). Perhaps, the reduction in functional prolactin receptors in NULL calves disrupted an unknown developmental or positive feedback mechanism that enhances prolactin secretion. The failure of NULL calves to mount a seasonal rise in prolactin also suggests that functional prolactin receptor signaling may be necessary for the positive feedback mechanisms that amplify prolactin secretion in response to increasing photoperiod.

SLICK calves had increased average daily gains and dry matter intakes during the feed efficiency study than WT calves and were also less feed efficient. Heat stress reduces voluntary feed intake (31, 32), and it is likely that SLICK calves were not as affected by heat stress, which allowed them to eat more, which increased growth rate and reduced feed efficiency. Increased dry matter intake is correlated with reduced feed efficiency (33), presumably because the resultant increased rate of passage through the digestive tract decreases nutrient absorption (34). In contrast to the beneficial effect of the SLICK mutations on growth in the feed efficiency trial, cattle inheriting a NULL mutation experienced reduced body weight and lower average daily gain than WT calves. The most likely explanation is that these calves had increased sensitivity to heat stress as compared with WT calves because of the heavy hair coats.

A heretofore unanswered question about cattle inheriting slick mutations in *PRLR* is whether they experience circannual rhythms in hair growth. While not completely addressed in the current experiment, the fact that hair weights of SLICK calves were heavier in the winter and early spring than in the summer is indicative that hair growth remains under seasonal control in these animals.

In conclusion, the genotype for *PRLR* has important effects on the ability of cattle for growth. Inheritance of mutations causing the slick phenotype led to increased growth during August to October, whereas knockout of a single allele of the gene reduced growth. These effects are most likely related to differences in hair growth and the ability to regulate body temperature during hot weather. Results strengthen the idea that cattle productivity in hot climates can be enhanced by incorporation of slick mutations in *PRLR* into cattle populations.

## Data Availability

The raw data supporting the conclusions of this article will be made available by the authors, without undue reservation.
