# Supplementary material for: Impact of mutations in PRLR for hair weight, circulating prolactin concentrations, feed intake, and growth of beef cattle in a hot and humid climate
Source: Front Endocrinol (Lausanne). 2026 Jul 24;17:1907038. doi: 10.3389/fendo.2026.1907038 (PMC13447229; doi:10.3389/fendo.2026.1907038)
Supplement: Supplementary file 1 [file Table1.docx]

**Supplemental Table 1**. Dietary composition and analyzed chemical composition of the diet fed during the feed efficiency trial.

|  | Basal diet |
| --- | --- |
| Item | Percent of dry matter |
| *Ingredients* |  |
| Corn gluten feed, pelleted | 50.00 |
| Soybean hulls, pelleted | 20.00 |
| Corn silage | 20.00 |
| Ground bermudagrass hay | 5.00 |
| Supplement^a^ | 5.00 |
| *Nutrient composition^b^* |  |
| DM, % | 68.9 |
| CP, %DM | 14.3 |
| EE, %DM | 3.03 |
| NDF, %DM | 42.4 |
| peNDF, %DM | 16.8 |
| Lignin, %DM | 2.33 |
| Ash, %DM | 6.36 |
| Starch, %DM | 18.12 |
| TDN, % | 68.9 |
| NE_m_, Mcal/kg | 1.67 |
| NE_g_, Mcal/kg | 1.08 |

^b^Pelleted supplement custom-formulated to provide vitamins (A, D, E) and minerals, and to supply 35 mg of monensin and 10 mg of thiamine per kg of diet DM (Furst-McNess Company, Freeport, IL).^c^DM = dry matter; CP = crude protein; EE = ether extract; NDF = neutral detergent fiber; peNDF: physically effective NDF, proportion of NDF that is effective in stimulating rumination and rumen mat formation, and is defined as the percent remaining on a 8 mm screen after sieving; TDN = total digestible nutrients calculated as: (CP digestibility × CP) + (FA × 2.25) + 0.98 × (100 – CP – Ash – EE) – 7; NE_m_ = net energy of maintenance; NE_g_ = net energy of gain.
